# Serial femtosecond crystallography datasets from G protein-coupled receptors

**DOI:** 10.1038/sdata.2016.57

**Published:** 2016-08-01

**Authors:** Thomas A. White, Anton Barty, Wei Liu, Andrii Ishchenko, Haitao Zhang, Cornelius Gati, Nadia A. Zatsepin, Shibom Basu, Dominik Oberthür, Markus Metz, Kenneth R. Beyerlein, Chun Hong Yoon, Oleksandr M. Yefanov, Daniel James, Dingjie Wang, Marc Messerschmidt, Jason E. Koglin, Sébastien Boutet, Uwe Weierstall, Vadim Cherezov

**Affiliations:** 1 Center for Free-Electron Laser Science, Deutsches Elektronen-Synchrotron DESY, Notkestraße 85, 22607 Hamburg, Germany; 2 School of Molecular Sciences, and Center for Applied Structural Discovery, Biodesign Institute, Arizona State University, Tempe, Arizona 85287-1604, USA; 3 Departments of Chemistry and Physics & Astronomy, The Bridge Institute, University of Southern California, Los Angeles, California 90089, USA; 4 MRC Laboratory of Molecular Biology, Francis Crick Avenue, Cambridge Biomedical Campus, Cambridge CB2 0QH, UK; 5 Department of Physics, Arizona State University, Tempe, Arizona 85287, USA; 6 Paul Scherrer Institute, Villigen CH-5232, Switzerland; 7 iHuman Institute, ShanghaiTech University, 2F Building 6, 99 Haike Road, Pudong New District, Shanghai 201210, China; 8 National Science Foundation BioXFEL Science and Technology Center, 700 Ellicott Street, Buffalo, New York 14203, USA; 9 Linac Coherent Light Source (LCLS), SLAC National Accelerator Laboratory, Menlo Park, California 94025, USA

**Keywords:** Nanocrystallography, G protein-coupled receptors, Biophysics

## Abstract

We describe the deposition of four datasets consisting of X-ray diffraction images acquired using serial femtosecond crystallography experiments on microcrystals of human G protein-coupled receptors, grown and delivered in lipidic cubic phase, at the Linac Coherent Light Source. The receptors are: the human serotonin receptor 2B in complex with an agonist ergotamine, the human δ-opioid receptor in complex with a bi-functional peptide ligand DIPP-NH_2_, the human smoothened receptor in complex with an antagonist cyclopamine, and finally the human angiotensin II type 1 receptor in complex with the selective antagonist ZD7155. All four datasets have been deposited, with minimal processing, in an HDF5-based file format, which can be used directly for crystallographic processing with CrystFEL or other software. We have provided processing scripts and supporting files for recent versions of CrystFEL, which can be used to validate the data.

## Background & Summary

Over the last few years, two X-ray free-electron laser (XFEL) facilities, the Linac Coherent Light Source (LCLS) and the SPring-8 Ångstrom Compact free-electron Laser (SACLA), have been put to extensive use in studying biological systems. The intense X-ray pulses outrun the conventional processes of radiation-induced damage, allowing diffraction data to be recorded from micron-sized crystals, while at the same time applying radiation doses far in excess of what would normally be considered tolerable in macromolecular X-ray crystallography using a laboratory or synchrotron source, even at cryogenic temperatures^1,2^. In turn, this allows data to be collected at near room temperature. Structure determination of G-protein-coupled receptors (GPCRs) represents a particularly successful application of serial femtosecond crystallography (SFX) at XFELs^3–6^. An injection device for viscous media^4^ has been recently developed, which allows microcrystals to be delivered to the X-ray beam in the lipidic cubic phase (LCP) medium in which they were grown, greatly simplifying the diffraction experiment by circumventing the need to extract and mount crystals.

**Figure 1:**
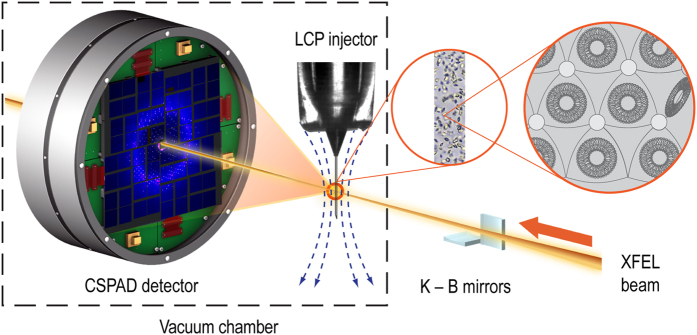
Schematic diagram of an LCP-SFX experiment. The stream of crystal-containing LCP is extruded from the LCP injector into the focus of the X-ray beam, formed by upstream optics (Kirkpatrick-Baez mirrors in this case). The LCP stream is kept straight by a coaxially flowing gas stream, indicated by the curved blue dashed lines. The X-ray beam is represented by the orange cylinder. Diffracted X-rays are measured by the detector (far left). The unscattered X-ray beam passes through a hole in the center of the detector. The LCP injector and CSPAD detector are located inside a sample vacuum chamber (dotted line). Adapted from (ref. 3).

This paper reports the deposition of four SFX datasets acquired from GPCRs using LCP-SFX (Fig. 1). The first dataset of the serotonin receptor 2B (5-HT_2B_) in complex with ergotamine was used to introduce the LCP-SFX technique^3^. In the original publication, a comparison was made between electron densities obtained by LCP-SFX at room temperature and conventional crystallography at cryogenic temperature using a synchrotron radiation source. This comparison showed small differences between the structures, attributed mostly to cryo-cooling, illustrating the importance of room temperature data collection. The second dataset of the smoothened receptor (SMO) in complex with cyclopamine was collected as part of an initial demonstration of the LCP injection technology^4^. SMO is involved in embryonal and stem cell development, and therefore considered as a promising anti-tumour target. The third dataset was for the δ-opioid receptor (δ-OR) in complex with a bifunctional peptide ligand DIPP-NH_2_, which acts as an agonist at μ-opioid and as an antagonist at δ-opioid receptors^5^. Such bi-functional ligands show promise as powerful analgesics with reduced propensity to inducing tolerance and dependency. The final dataset is of the angiotensin II type 1 receptor (AT_1_R) in complex with the selective antagonist ZD7155 (ref. 6). AT_1_R is a primary regulator of blood pressure in the human body and the target for treating hypertension.

## Methods

The preparation of samples, data acquisition and processing for all four GPCRs have been described previously^3–6^. GPCR microcrystals were prepared using the following general protocols, and specific details, and small deviations from these protocols are noted under particular target receptor sections below.

All engineered for crystallization GPCR constructs (Table 1) were cloned into a modified pFastBac vector (Invitrogen) cassette containing the necessary tags, and expressed in *Spodoptera frugiperda* (*Sf9*) insect cells for 48 h at 27 °C using recombinant baculovirus at a MOI (multiplicity of infection) of 5. Cells were lysed by osmotic pressure and total membranes were isolated and purified by repeated Dounce homogenization and centrifugation in hypotonic and hypertonic buffers. GPCR-ligand complexes were subsequently formed by incubating purified membranes in the presence of the respective ligand, iodoacetamide and EDTA-free protease inhibitor cocktail, followed by extraction of the complexes in N-dodecyl-β-D-maltopyranoside (DDM, Anatrace) and cholesteryl hemisuccinate (CHS, Sigma) mixture. Solubilized receptors were purified by immobilized metal affinity chromatography (IMAC) using TALON (Clontech) resin and concentrated to ~20–50 mg ml^−1^. Crystallization was performed in Hamilton gas-tight syringes as previously described^7^. Briefly, purified receptors were first reconstituted in LCP by mixing protein solution with monoolein doped with 10% w/w cholesterol at a 2:3 (v/v) ratio using a syringe mixer. Approximately 5 μl of protein-laden LCP were carefully injected as a continuous column ~400 μm in diameter into each 100 μl Hamilton syringe filled with 60 μl of the corresponding precipitant solution (Table 2) and incubated at 20 °C until crystals formed. Just prior to starting data collection, samples from several syringes were consolidated together, the excess precipitant solution was carefully removed, followed by the addition of ~25% v/v of 7.9 MAG to absorb the residual precipitant solution. The crystal samples were characterized on site at LCLS by optical and UV fluorescence imaging as well as by SONICC^8^.

**Figure 2:**
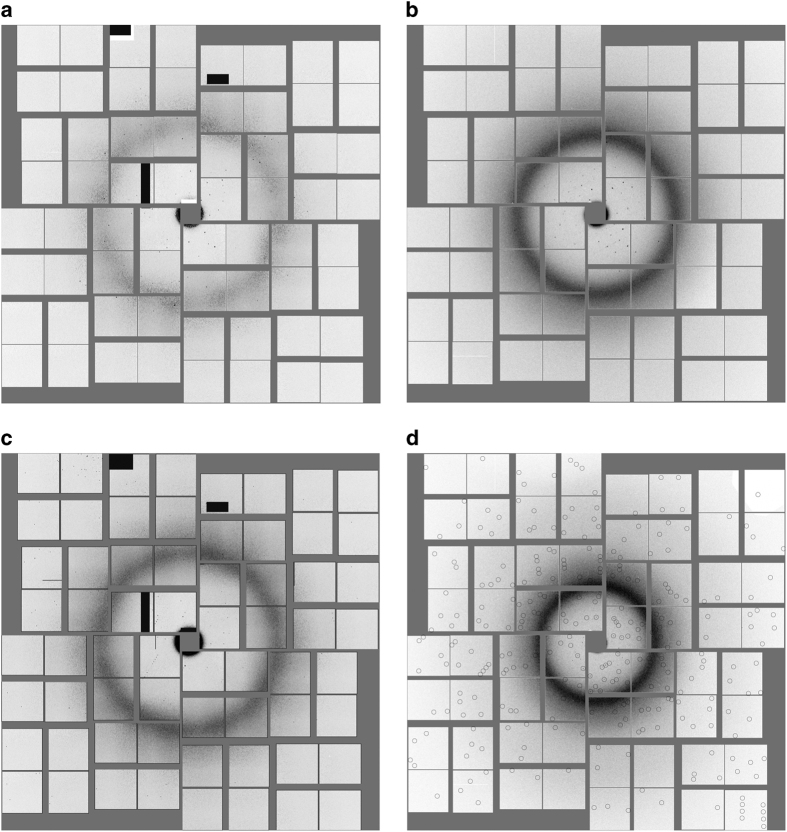
Representative diffraction patterns from (**a**) 5-HT_2B_, (**b**) AT_1_R, (**c**) SMO and (**d**) δ-OR. The solid grey areas are the regions outside the panels of the detector. The diffuse circle arises from the LCP matrix, and sharp spots from the crystals. Pattern (**d**) is shown with calculated reflection positions circled, but (**a**–**c**) are not.

Diffraction patterns (Fig. 2) were acquired at the Coherent X-ray Imaging (CXI) endstation at LCLS using a lipidic cubic phase injector with 50 μm nozzle operating at a flow rate of 150–200 nl min^−1^ (ref. 4). X-rays were focused using Kirkpatrick-Baez mirrors to create a focal spot of about 1.5 μm diameter, with wavelengths as stated below. The diffraction patterns were recorded on a Cornell-SLAC Pixel Array Detector (CSPAD)^9,10^. The detector data, as well as data from a wide range of other detectors such as X-ray pulse energy monitors and electron bunch measurements (used to calculate the wavelength of each X-ray pulse, which varied by a small amount due to the Self-Amplified Spontaneous Emission (SASE) process of the LCLS), were all stored by the LCLS data acquisition system in Extensible Tagged Container (XTC) files^11^. These files are extremely large because they contain all readouts from every X-ray pulse, regardless of whether or not the X-ray pulse hit a crystal. Most pulses did not hit crystals, as shown by the ‘hit rates’ in Table 3. For further processing, we used Cheetah^12^ to identify ‘hits’ (images containing a pre-set minimal number of Bragg reflections) and assembled the detector data, after minimal detector corrections, to HDF5 files^13^. Relevant information such as the X-ray wavelengths, pulse energies and timestamps, was also written to the HDF5 files. The HDF5 files have been deposited to the Coherent X-ray Imaging Data Bank (CXIDB). The differences in processing parameters for Cheetah are summarised in Table 4. The differences are in most cases unimportant to the interpretation of the data and represent historical developments in Cheetah (for example, the change to a radial method of background subtraction). For δ-OR and AT_1_R, the peak search area was restricted to avoid artefacts visible near the edges of the detector.

### Serotonin receptor, 5-HT_2B_

Purified membranes from *Sf9* cells that expressed engineered 5-HT_2B_ receptor (Table 1) were incubated in the presence of 100 μM Ergotamine for 1 h at room temperature (21–23 °C). Extraction of the complexes was done using 1% (w/v) DDM/0.2% (w/v) CHS. At the final purification stage, the sample buffer was exchanged into 50 mM HEPES pH 7.5, 150 mM NaCl, 0.05% (w/v) DDM, 0.01% (w/v) CHS, 10% (v/v) glycerol, and 50 μM ergotamine, and the protein was concentrated to~20 mg ml^−1^. Microcrystals with an average size of 5×5×5 μm were obtained in syringes within 24 h at 20 °C and used for SFX data collection.

The X-ray wavelength for data acquisition was 1.3 Å (photon energy 9.5 keV) and data were acquired at sample-detector distances of 82, 112 and 152 mm (the majority). The 5-HT_2B_ data collection was performed during LCLS experiment L672 in March 2013.

Data from LCLS in facility XTC format were processed using Cheetah. A total of 4,217,508 detector frames were processed, from which Cheetah found 152,531 crystal hits (Table 4). The detector distance was read from EPICS process variable CXI:DS1:MMS:06.RBV. Frames corresponding to a hit were saved to individual HDF5 data files with only detector corrections applied (in this case dark offset subtraction and a per-pixel gain correction derived from flat field data). Processing parameters and calibration files are archived with the deposited data for reference.

Our new indexing and integration with CrystFEL 0.6.2 was performed using the same parameters as for the original processing, as follows: peak search mode ‘zaef’ with threshold 450, minimum squared gradient 500 and minimum SNR (signal-to-noise) 4. Indexing was performed with Mosflm 7.2.0 (using prior unit cell parameters and lattice type information), followed by DirAx, and finally Mosflm without any prior information as a final fallback. The target unit cell was primitive orthorhombic with a=61.7 Å, b=122.8 Å and c=168.1 Å. Indexing solutions were accepted if the reciprocal cell axis lengths and angles matched within 4% of the target lengths and 1.4° of the target angles respectively. Reflections were integrated using shoebox summation integration with an inner radius of 3 pixels and a background annulus between 7 and 8 pixels centred on each calculated reflection position.

### Smoothened receptor

Purified membranes from *Sf9* cells that expressed engineered SMO (Table 1) were incubated for 1 h at 4 °C in the presence of 50 μM of antagonist cyclopamine, followed by extraction of the receptor/ligand complexes in 1% (w/v) DDM and 0.2% (w/v) CHS. The protein was eluted in a buffer containing 50 mM HEPES, pH 7.5, 300 mM NaCl, 10% (v/v) glycerol, 0.03% (w/v) DDM, 0.006% (w/v) CHS, 250 mM imidazole and 50 μM ligand and concentrated to 50 mg ml^−1^ for crystallization. Microcrystals with maximal size less than 5 μm were obtained in syringes and used for SFX data collection.

The X-ray wavelength for data acquisition was 1.3 Å (photon energy 9.5 keV) and data were acquired at sample-detector distances of 121 and 151 mm (the majority). The SMO data collection was performed during LCLS experiment L672 in March 2013.

Data from LCLS in facility XTC format were processed using Cheetah. A total of 3,510,525 detector frames were processed, from which Cheetah found 274,214 crystal hits (Table 4). The detector distance was read from EPICS process variable CXI:DS1:MMS:06.RBV. Frames corresponding to a hit were saved to individual HDF5 data files with only detector corrections applied (in this case dark offset subtraction and a per-pixel gain correction derived from flat field data). Processing parameters and calibration files are archived with the deposited data for reference.

Our new indexing and integration using CrystFEL 0.6.2 was performed using the same parameters as for the previous processing, as follows: peak search mode ‘zaef’ with threshold 600, minimum squared gradient 3,000 and minimum SNR 6. Indexing was performed with Mosflm 7.2.0 (using prior unit cell parameters and lattice type information), followed by DirAx, and finally Mosflm without any prior information as a final fallback. The target unit cell was primitive monoclinic with a=40.5 Å, b=157.3 Å, c=52.4 Å and β=97°. Indexing solutions were accepted if the reciprocal cell axis lengths and angles matched within 7% of the target lengths and 6° of the target angles respectively. Reflections were integrated using shoebox summation integration with an inner radius of 3 pixels and a background annulus between 4 and 5 pixels centred on each calculated reflection position.

### Delta-opioid receptor

Purified membranes from *Sf9* cells that expressed engineered δ-OR (Table 1) were incubated for 1 h at 4 °C in the presence of 50 μM of bi-functional peptide ligand DIPP-NH_2_, followed by extraction of receptor/ligand complexes in 0.75% (w/v) DDM/0.15% (w/v) CHS and purification using IMAC. After purification the protein was treated overnight with histidine-tagged TEV protease to cleave the N-terminal histidine tag and Flag tag. TEV protease and the cleaved N-terminal fragment were removed by incubation with TALON resin for 1 h at 4 °C. Purified receptor in 50 mM HEPES, pH 7.5, 500 mM NaCl, 10% (v/v) glycerol, 0.03% (w/v) DDM, 0.006% (w/v) CHS and 50 mM DIPP-NH_2_ was concentrated to 40 mg ml^−1^. Purified δ-OR-DIPP-NH_2_ complex was reconstituted in LCP as described above. Microcrystals with average size of 5×2×2 μm were obtained in syringes and used for SFX data collection.

The X-ray wavelength for data acquisition was 1.6 Å (photon energy 7.95 keV) and data were acquired at sample-detector distances of 78.8 mm (the majority) and 109 mm. The δ-OR data collection was performed during LCLS experiment LA25 in January 2014.

Data from LCLS in facility XTC format were processed using Cheetah. A total of 1,967,539 detector frames were processed, from which Cheetah found 125,458 crystal hits (Table 4). Frames corresponding to a hit were saved to individual HDF5 data files with only detector corrections applied (in this case only dark offset subtraction). Processing parameters and calibration files are archived with the deposited data for reference.

Our new indexing and integration using CrystFEL 0.6.2 was performed using the same parameters as for the previous processing, as follows: the Bragg peaks found by Cheetah during the hit-finding stage were used for indexing with Mosflm 7.2.0 (using prior unit cell parameters and lattice type information), followed by DirAx, and finally Mosflm without any prior information as a final fallback. The target unit cell was monoclinic C with a=156.23 Å, b=89.29 Å, c=96.42 Å and β=92.3°. Indexing solutions were accepted if the reciprocal cell axis lengths and angles matched within 5% of the target lengths and 1.5° of the target angles respectively (the default values in CrystFEL 0.6.2). Reflections were integrated using shoebox summation integration with an inner radius of 3 pixels and a background annulus between 4 and 5 pixels centred on each calculated reflection position.

### Angiotensin receptor

Purified membranes from *Sf9* cells that expressed engineered AT_1_R (Table 1) were incubated for 1 h at 4 °C in the presence of 100 μM of the antagonist ZD7155, followed by extraction of the receptor/ligand complexes in 1% (w/v) DDM/0.2% (w/v) CHS. After purification by IMAC the receptor sample was desalted to remove imidazole using PD MiniTrap G-25 column (GE Healthcare) and then treated overnight with His-tagged TEV protease to cleave the N-terminal FLAG/His tags from the protein. The cleaved FLAG/His tags and TEV protease were removed by incubation with TALON resin. The protein was not treated with PNGase F and therefore remained fully glycosylated. Finally, the purified protein was concentrated to 30 mg ml^−1^ and used for crystallization. Microcrystals with an average size of 10×2×2 μm were obtained in gas-tight Hamilton syringes and used for SFX data collection.

The X-ray wavelength for data acquisition was 1.6 Å (photon energy 7.95 keV) and the sample-detector distance was 108 mm. The AT_1_R data collection was performed during LCLS experiment LA25 in January 2014.

Data from LCLS in facility XTC format were processed using Cheetah. A total of 2,764,739 detector frames were processed, from which Cheetah found 457,275 crystal hits (Table 4). Frames corresponding to a hit were saved to individual HDF5 data files with only detector corrections applied (in this case only dark offset subtraction). Processing parameters and calibration files are archived with the deposited data for reference.

Our new indexing and integration using CrystFEL 0.6.2 was performed using the same parameters as for the previous processing, as follows: the peaks found by Cheetah during the hit-finding stage were used for indexing with Mosflm 7.2.0 (using prior unit cell parameters and lattice type information), followed by DirAx, and finally Mosflm without any prior information as a final fallback. The target unit cell was monoclinic C with a=72.8 Å, b=41.0 Å, c=167.7 Å and β=99.4°. Indexing solutions were accepted if the reciprocal cell axis lengths and angles matched within 5% of the target lengths and 1.5° of the target angles respectively (the default values in CrystFEL 0.6.2). Reflections were integrated using shoebox summation integration with an inner radius of 3 pixels and a background annulus between 4 and 5 pixels centred on each calculated reflection position.

### Code availability

The hit-finding and processing program Cheetah is free and open source software distributed under the GNU General Public Licence version 3 (GPL3), and may be downloaded from the following web location: https://www.desy.de/~barty/cheetah.

## Data Records

We have deposited four datasets, one for each of the receptors: 5-HT_2B_ (Data Citation 1), SMO (Data Citation 2), δ-OR (Data Citation 3) and AT_1_R (Data Citation 4), as shown in Table 3. Out of the millions of frames acquired, each dataset consists of the ‘hits’, where the X-ray pulse intersected a crystal and gave rise to Bragg peaks as determined by Cheetah (Fig. 2). This reduces the volume of data by a factor of about 20, to a level, which is practical for download and processing, while retaining practically all of the useful information. Only minimal detector corrections have been applied, namely the subtraction of detector ‘dark’ signal determined using frames acquired just before or after with the X-ray beam shutter closed, and a per-pixel gain correction for 5-HT_2B_ and SMO.

The datasets have been deposited in a format based on the Hierarchical Data Format, version 5 (HDF5). HDF5 is a ‘container format’ which has some of the properties of a file system, allowing arbitrary numbers of arrays, each of arbitrary dimensionality, to be contained in ‘groups’ which behave like directories and can be nested to form a tree structure. This format allows the image data to be stored alongside the X-ray photon energy (as estimated from the electron beam and undulator parameters), pulse energy, motor positions relating to the sample-detector distance, and so on. HDF5 makes the data ‘self-describing’ as far as the identification of datasets is concerned, for example the photon energy is found at ‘/LCLS/photon_energy_eV’ and the image data itself at ‘/data/rawdata’. This makes the data very much easier to handle compared to the raw data format output by the LCLS data acquisition system, which is an ‘XTC’ (Extensible Tagged Container) file containing readouts from all detectors and sensors, and cannot easily be interpreted without using facility-specific software which is not supported for use outside the LCLS computing environment. Nevertheless, some extra information is required to map the pixel coordinates into physical space and hence interpret the data. We have provided this information in the form of CrystFEL geometry files. The syntax of CrystFEL geometry files is described fully in the CrystFEL documentation.

## Technical Validation

All four datasets have previously been indexed, integrated and merged using CrystFEL, a software suite created specifically for processing serial crystallography data^14^. The resulting merged data have been used to solve the structures of the corresponding receptor complexes by molecular replacement, as previously described^3–6^. To validate the data, we have repeated the indexing, integration and merging stages using the latest version of CrystFEL, version 0.6.2. We did not further refine the detector geometry, but rather used the previous detector geometry files after a small amount of conversion to render them compatible with the latest version of CrystFEL. Merging was performed using *partialator* from CrystFEL, without partiality modelling (partialator's ‘unity’ model), three iterations of scaling and merging, excluding reflections with any pixel intensity exceeding 14,000 detector units. The intensities were merged according to point group mmm for 5-HT_2B_ and 2/m (unique axis b) for AT_1_R, δ-OR and SMO. The default values were used for all other parameters. Per-pattern resolution cutoffs, in which intensities were merged up to 1 nm^−1^ above the resolution estimate based on the limit of the found peaks, were used for two of the data sets (δ-OR and AT_1_R) but not the others. The CrystFEL processing parameters for the updated results are summarised in Table 5. The differences in peak search methods are simply because more recent versions of Cheetah were used for two of the data sets (δ-OR and AT_1_R), and the more recent versions are able to find peaks more effectively than CrystFEL's internal algorithm. Differences in peak search parameters are because of differences in the scattering strength of the crystals and background levels. The unit cell parameter tolerances must be wide enough to capture all the indexing solutions which match the target parameters, while excluding other indexing solutions. In practice, a rather generous tolerance can safely be used for this, as was the case for SMO.

**Figure 3:**
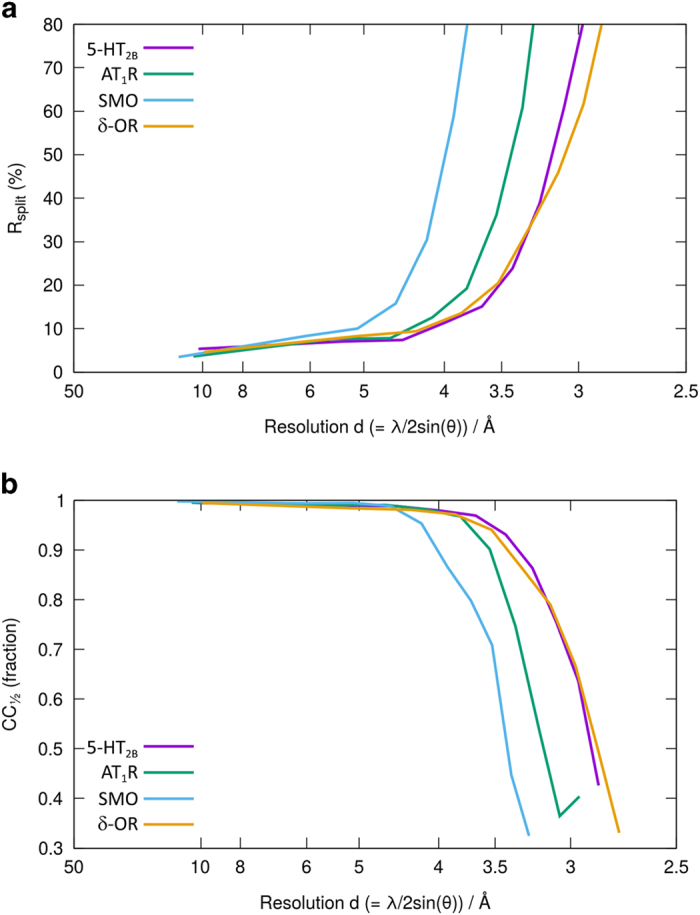
Plots of the self-consistency figures of merit (**a**) R_split_ and (**b**) CC_1/2_ against resolution for all four datasets as re-processed here.

**Figure 4:**
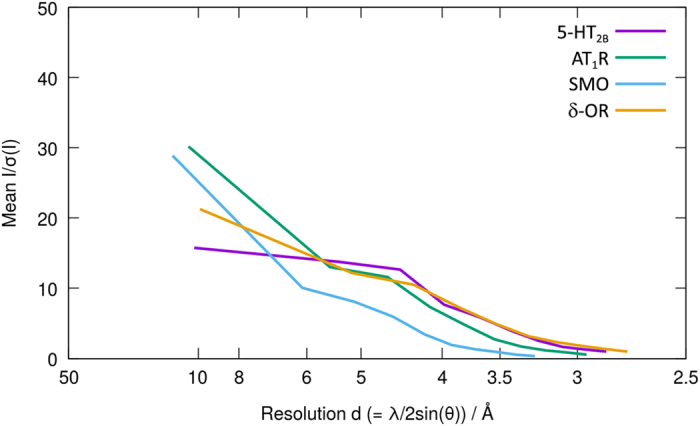
Plots of the signal-to-noise ratio I/sigma(I) for all four datasets as reprocessed here.

We calculated the usual figures of merit for SFX data (Rsplit, CC* and I/sigma(I)) over the same resolution ranges used for the original publications (Figs 3 and 4). Although we have not repeated the full structure determination, the figures of merit calculated during processing the data are almost all better than the previously published results, even despite a lower number of indexed patterns used in two of the cases. The lower number of indexed patterns is most likely due to the additional processing stages, which have been added in recent versions of CrystFEL. These new processing routines impose additional restrictions on the quality of the data resulting in rejection of some patterns^15^. The figures of merit are shown in Table 6.

## Usage Notes

The deposited data can be processed directly using CrystFEL. For each dataset, we have deposited a script, called ‘run-processing’, which contains short series of commands necessary to index, integrate and merge the data, and to produce the figures of merit shown in Table 6. In addition, we have deposited an archive of CrystFEL 0.6.2 in source code form with each dataset.

## Additional Information

**How to cite this article:** White, T. A. *et al.* Serial femtosecond crystallography datasets from G protein-coupled receptors. *Sci. Data* 3:160057 doi: 10.1038/sdata.2016.57 (2016).

## Supplementary Material



## Figures and Tables

**Table 1:** Summary of the receptor constructs used for structure determination.

**Receptor/ligand**	**N-term tags**	**N-term truncations**	**C-term tags**	**C-term truncations**	**Mutations**	**Fusion partner**
5-HT_2B_/ergotamine	HA-Flag	1–35	PP-10 × His	406–481	M144W	BRIL in ICL3 (T249 to V313)
SMO/cyclopamine	HA-Flag-10 × His-TEV	CRD (1–189)	None	556–787	None	BRIL in ICL3 (P434 to K440)
δ-OR/DIPP-NH_2_	HA-Flag-10 × His-TEV	1–38	None	339–372	None	N-term BRIL
AT_1_R/ZD7155	HA-Flag-10 × His-TEV	1, 7–16	None	320–359	None	N-term BRIL
10 × His, 10-mer His tag; BRIL, thermostabilized apocytochrome b562RIL from *Escherichia coli* (M7W, H102I and R106L); C-term, C terminal; CRD, cysteine-rich domain; Flag, flag tag; HA, hemagglutinin signal sequence; ICL3, intracellular loop 3; N-term, N terminal; PP, prescission protease-recognition site; TEV, TEV protease-recognition site.						

**Table 2:** Summary of crystallization conditions.

**Receptor/ligand**	**Buffer**	**Precipitant**	**Salt**	**Additive**
5-HT2B/ergotamine	0.1 M Tris pH 8.0	30% v/v PEG 400	20–80 mM MgCl_2_	
SMO/cyclopamine	0.1 M Hepes pH 7.0	30% v/v PEG 400	100 mM NaCl	
δ-OR/DIPP-NH_2_	0.1 M MES pH 6.0	30-32% v/v PEG 400	50–180 mM Li citrate	
AT_1_R/ZD7155	0.1 M Na Citrate pH 5.0	28% v/v PEG 400	450 mM NH_4_H_2_PO_4_	4% v/v DMSO

**Table 3:** Basic information about the four datasets.

**Receptor/ligand**	**Data citation**	**CXIDB accession number**	**PDB accession code for XFEL structure**	**Acquisition dates**	**Number of frames acquired**	**Number of hits found (overall hit rate)**
5-HT_2B_/ergotamine	1	21	4NC3	20–24 March 2013	4,217,508	152,531 (3.6%)
SMO/cyclopamine	2	39	4O9R	20–24 March 2013	3,510,525	274,214 (7.8%)
δ-OR/DIPP-NH_2_	3	40	4RWD	2–3 Feb 2014	1,967,539	125,458 (5.9%)
AT_1_R/ZD7155	4	38	4YAY	31 Jan-2 Feb 2014	2,764,739	457,275 (17%)

**Table 4:** Parameters for hit finding using Cheetah.

**Receptor/ligand**	**Minimum number of peaks**	**Peak criteria**	**Peak search radii (pixels)**	**Cheetah peakfinder algorithm**	**Background subtraction for peak search**
5-HT_2B_/ergotamine	15	2–20 pixels above 180 ADU	75–3,000	3	Median filter, 3 pixels radius
SMO/cyclopamine	15	2–20 pixels above 180 ADU	75–3,000	3	Median filter, 2 pixels radius
δ-OR/DIPP-NH_2_	15	2–40 pixels above 40 ADU with SNR above 4	70–700	8	Radial background subtraction
AT_1_R/ZD7155	15	2–40 pixels above 40 ADU with SNR above 4	70–700	8	Radial background subtraction

**Table 5:** Parameters for data processing using CrystFEL.

**Receptor/ligand**	**Peak search method**	**Peak search parameters**	**Unit cell tolerance**	**Peak integration radius (pixels)**	**Radii of background annulus (pixels)**	**Per-crystal resolution cutoff**
5-HT_2B_/ ergotamine	CrystFEL internal (‘zaef’)	Threshold 450, min grad 500, SNR 4	4%, 1.4°	3	7–8	No cutoff
SMO/ cyclopamine	CrystFEL internal (‘zaef’)	Threshold 600, min grad 3000, SNR 6	7%, 6°	3	4–5	No cutoff
δ-OR/DIPP-NH_2_	Peaks from Cheetah	See Table 4	5%, 1.5°	3	4–5	1.0 nm^−1^ above conservative resolution estimate
AT_1_R/ZD7155	Peaks from Cheetah	See Table 4	5%, 1.5°	3	4–5	1.0 nm^−1^ above conservative resolution estimate

**Table 6:** Crystallographic statistics of the four datasets from re-processing using CrystFEL 0.6.2.

**Receptor/ ligand**	**Number of crystals found**	**Number of crystals merged**	**Resolution range, Å**	**R**_**split**_ **, %**	**CC** ^*^	**I/sigI**
5-HT_2B_/ ergotamine	20,589 [32,819]	17,860 [32,819]	35–2.8 (2.9–2.8)	6.71 (116) [9.5 (162)]]	0.9990 (0.773) [0.998 (0.74)]	6.73 (0.99) [5.9 (0.64)]
SMO/ cyclopamine	42,640 [61,964]	42,198 [61,964]	40–3.2 (3.3–3.2)	8.94 (385) [9.8 (63.2)]	0.9995 (0.7004) [0.9991 (0.28)]	6.20 (0.34) [7.4 (1.8)]
δ-OR/DIPP-NH_2_	70,899 [36,083]	70,767 [36,083]	33.5–2.7 (2.8–2.7)	11.9 (121) [11.8 (87.9)]	0.9980 (0.7053) [Not reported]	6.63 (0.989) [6.0 (1.3)]
AT_1_R/ZD7155	79,558 [73,130]	77,282 [71,130]	32.6–2.9 (3.0–2.9)	8.60 (209) [9.8 (140)]	0.9990 (0.7587) [0.999 (0.872)]	7.60 (0.55) [8.2 (0.84)]
Values in parentheses are those for the highest resolution shell. Values in square brackets are those from the previously reported processing using old CrystFEL versions.						
